# Association of *Helicobacter pylori* Infection and Host Cytokine Gene Polymorphism with Gastric Cancer

**DOI:** 10.1155/2021/8810620

**Published:** 2021-05-28

**Authors:** Md. Zeyaullah, Abdullah M. AlShahrani, Irfan Ahmad

**Affiliations:** ^1^Department of Basic Medical Science, College of Applied Medical Sciences, King Khalid University (KKU), Khamis Mushait Campus, Abha, Saudi Arabia; ^2^Genomic Science Academy, Muzaffarpur, Bihar, India; ^3^Department of Biosciences, Jamia Millia Islamia (A Central University), New Delhi, India

## Abstract

The global cancer burden of new cases of various types rose with millions of death in 2018. Based on the data extracted by GLOBOCAN 2018, gastric cancer (GC) is the third leading cause of mortality related to cancer across the globe. Carcinogenic or oncogenic infections associated with *Helicobacter pylori* (Hp) are regarded as one of the essential risk factors for GC development. It contributes to the increased production of cytokines that cause inflammation prior to their growth in the host cells. Hp infections and specific types of polymorphisms within the host cells encoding cytokines are significant contributors to the hostʼs increased susceptibility in terms of the development of GC. Against the backdrop of such an observation is that only a small portion of the cells infected can become malignant. The diversities are a consequence of the differences in the pathogenic pathway of the Hp, susceptibility of the host, environmental conditions, and interplay between these factors. It is evident that hosts carrying cytokine genes with high inflammatory levels and polymorphism tend to exhibit an increased risk of development of GC, with special emphasis being placed on the host cytokines gene polymorphisms.

## 1. Introduction

*Helicobacter* is one of the genera of enteric microbes and has been known to reside within humansʼ gastric epithelial tissue. The genus contains various species of Gram-negative bacteria, including oncogenic *Helicobacter pylori* (Hp), which is one of the primary causes of gastric cancer (GC) [1, 2]. The stomach of the enteric system is the most suitable anatomical niche for colonization of Hp, which results in the development of chronic gastritis, ulcerative colitis, and ultimately the malignant transformation of the gastric mucosa with the inclusion of the mucosal-associated lymphoid tissue (MALT) lymphoma [3]. The oncogenic effects of this pathogen account for more than 75% of gastric malignancy [4]. By 2018, cancerʼs worldwide burden was projected to have risen to more than 18 million new infections with more than 9.5 fatalities [5].

Moreover, the projection of the prevalence within the coming years is approximated to be 43 million [5]. More than 1.7 million new cancer cases were to be reported in 2019, while over 606,880 cases of cancer-related deaths were expected by the end of 2019 based on “Cancer Facts & Figures 2019,” which is an estimation of about 1660 deaths per day [6]. The occurrence of stomach cancer has been on a declining trend in most nations globally over the past 70 years. Stomach cancer is regarded as one of the most common malignancies and one of the leading contributors to deaths related to cancer across the world [7, 8], with more than 50% of the worldʼs total cases being exhibited in eastern Asia and majorly in China [9]. The reasons behind the reductions in such enormous levels of mortalities related to GC are not fully documented, even though researchers have attributed the decline to the alteration of the lifestyle and dietary factors and steps towards the adoption of healthy living practices. Diets that are rich in adequate amounts of fruits and vegetables tend to provide anticancer secondary metabolites. This implies that the reason for regression in the incidence of GC is comprised of decreasing the levels of consuming salted and junk or foods with preservatives, increasing the levels of awareness regarding the consumption of adequate amounts of fruits and vegetables, which in turn inhibit the chronic infections of microbial carcinogens [10, 11].

According to existing evidence, people with gastric dysplasia, severe and extensive gastric atrophy, extensive gastric intestinal metaplasia, and incomplete subtype of intestinal metaplasia are at greater risk of developing GC. To try to identify these individuals, a variety of noninvasive, endoscopic, histological, or combined approaches may be used.

In western countries, the main approach is histology-based, while in eastern countries, with a high prevalence of GC, the main approach is endoscopy-based. Despite the fact that some interventions have demonstrated their cost effectiveness and potential to minimize GC mortality, many regions are unable to implement them due to resource constraints. An integrated and resource-sensitive approach for real-life practice could be built based on existing evidence from both western and eastern perspectives [12].

Just a few Asian countries with a greater risk of GC have implemented population-based screening systems so far. Preendoscopic risk assessment based on demographic and clinical characteristics, such as ethnicity, age, gender, smoking, and Hp status, may help identify subjects with a high pretest likelihood for a potentially cost-effective approach, particularly in intermediate- and low-risk countries. Endoscopy offers a real-time evaluation of the patientʼs risk level, so combining endoscopic and histological methods should always be considered. Furthermore, imaging enhanced endoscopy (IEE) has been shown to improve the association between endoscopic and histological findings by facilitating targeted biopsies [12].

**Figure 1:**
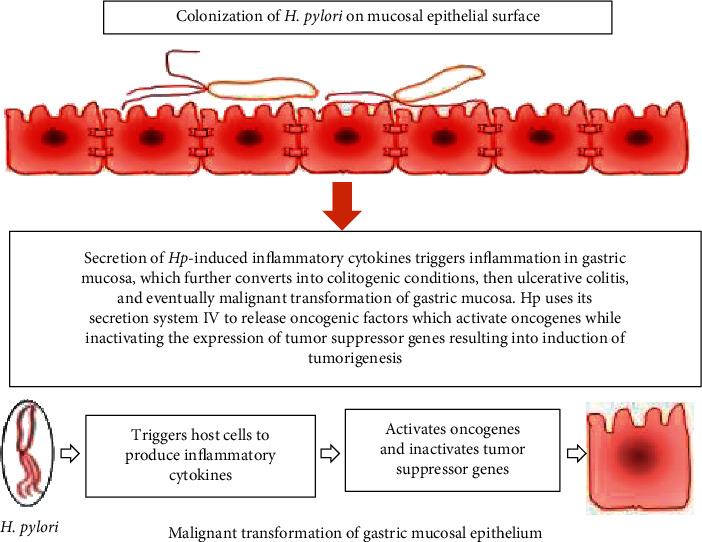
Hp-induced malignant transformation of gastric cancer (GC) in the human gut.

Hp-induced persistent chronic inflammation has been reported to be linked with epithelial dysplasia, and this precancerous stage further developed into GC following the oncogenic cascades [13, 14]. It is interesting to observe that inasmuch as the incidence of Hp contamination has a range of 40–80% among people, only a marginal proportion with a likelihood of less than 3% infected humans are reported to develop GC [3]. From the observation that Hp relies on the gastric mucosa of the host cell as an opportunistic pathogen, whenever it gets the opportunity to transform the epithelial cells, it depends on the oncogenic cascades targeting dysregulation of specific cytokines to cause cancer (Figure 1). Hp induced aberrant genetic mutations of the cytokines as well as the cell receptors encoding the genes, which in turn establishes the severity of the response of inflammation to the bacteria, and this, in turn, fuels the development of gastric lesions [15].

The cytokine IL-1*β* is one of the most important inflammasomes, which plays a crucial role in signaling mechanisms meant to modulate gastric tumorigenesis [16]. Furthermore, Hp-induced generation of reacting free radical species tends to promote malignant transformation of inflamed gastric epithelia [17]. Chronic infectious effect of Hp causes gastric inflammation, and consequences are synthesis and release of various proinflammatory cytokines of the gastric acid secretion as a result of the increased level of these cytokines. Thereafter, a process of pathogenesis occurs, and consequently, neoplastic growth of the gastric mucosa is followed by excessive secretion of gastrin and free radical generation [18]. The aberrant inflammatory response may be linked with malignant transformation of gastric mucosa [15]. Aberrant disruption of intact biofilm layered by enteric probiotics facilitates oncogenic bacteria to inflame mucosal epithelia [19].

The combined effect of hypergastrinemia, inflammasomes, pathobionts, and exogenous and endogenous oxidative stresses, including the host-derived oncogenic factors, tends to trigger tumorigenesis [16, 17, 19–21]. These multifactorial disorders forced the cellular genome to perform aberrant regulation of the cell cycle, leading to the genetic and epigenetic alteration that ultimately results in malignant transformations.

## 2. Genetic Diversity in *Helicobacter pylori*

Hp is characteristic in terms of its genetic diversity across the species [22, 23], nucleotide sequence diversity of the individual genes associated with the high rates of mutation, and high level of recombination across the species [24, 25]. The strains from different human hosts are distinct in many aspects, including the sequences of the specific genes, as they also exhibit variations in the contents of the genes and the organization of chromosomes. The primary genome of Hp is comprised of 1,100 genes that are exhibited in all strains, with each of the strains comprising of other hundreds of additional genes that are not currently present on the universal basis. Hp coexists with the host as normal flora and exhibits an aberrant evolution in specified oncogenic arsenals or secretion systems. The product of cytotoxin-associated gene A (CagA) of Hp contributes to the aberrant generation of reactive oxygen species (ROS), resulting in the activation of some mechanistic pathways of tumorigenesis [26–30]. The pathways for signaling are relegated to the Wnt or *β*-catenin, Ras, and Akt-mTOR. The pathogen is a naturally competent cell and has the potential of developing distinct type IV secretion systems (T4SS) as the primary comB-system that integrates with the DNA containing oxygen ions into its genome via the process of recombination of genes [31, 32]. As of now, there is a tremendous development to expose molecular cross-talk between Hp-induced activation of oncogenes/inactivation of tumor suppressor genes and CagA gene comprising of SSIV [1]. Aberrant regulation of the cell cycle of gastric epithelia following oncogenic Hp infection triggers sequential oncogenic pathways resulting in a malignant transformation of the gastric mucosa (Figure 1). The gastric section of the host is comprised of low diversity of bacteria on a specific level, while it is rich in terms of the genetic variants among the distinct subpopulations of the Hp. The preservation of high levels of diversification is an adaptive feature that allows the bacterium to resist possible challenges that might be presented in the individual hosts [33].

Within the past ten years, different researchers have been attracted to analyze the Hp isolates from humans having different states of diseases as they document evidence meant to identify the specific features of the strain that have a significant correlation with the presence of GC and the premalignant histologic lesions. The variations that are specific to the strain in the secreted proteins and proteins exposed at the surface have a significant correlation with the increase in the risk of GC [34].

### 2.1. Cytotoxin-Associated Gene A (CagA)

All carcinoma cells of GC act as hosts to the genome of Epstein-Barr virus (EBV) and act as an intragenomic parasite for the regulation of expression of the cagA gene of Hp [35]. From the analysis of its phenotype, the strain can be classified into different distinct types, including those containing the gene that is associated with the expression of cytotoxin, which is commonly referred to as the *CagA* or cytotoxin-associated gene A, and those that do not have the potentiality of cytotoxin expression [36, 37]. The Hp positive strain of *CagA* that is made up of the isolates from the United States were found to contribute to the high levels of gastric mucosa inflammation compared with the negative strains of *CagA* [38–42]. The positive strains of these bacteria that are known to be carriers of *cag PAI* infections were found to have a higher probability of progression into atrophic gastritis compared with the negative infections by the *CagA* [43]. Moreover, the nested case-control research performed on the Japanese-American males showed that *CagA* antibodies highly infect people having gastric malignancy compared with the population that did not have such a form of malignancy [44]. The most distinct variation among the strains of Hp from people that do not seem to be closely related is the presence or absence of the region of the chromosome called the cag pathogenicity island (PAI), which is considered as the marker for enhanced virulence. The *cagPAI* could be a DNA fragment that is 40 kb and is formed from approximately 30 genes that are flanked by 31-bp direct replications [45]. The DNA fragment is known to encode *CagA* because the major determinant of virulence of Hp plays an essential role in the host signaling pathways for triggering tumorigenesis and also the main entities found within the type IV section system [46, 47]. The fragment has the viability of dislocating *CagA* into the cells of the host animal [48]. Upon entry into the cells of the host individual, *CagA* binds to different host cell proteins for disruption of intracellular signaling systems through tyrosine phosphorylation-dependent or pathways that are independent [49]. This results in the elongation and loss of the polarity of the cells of the host individual, the promotion of its proliferation, and inflammation. The presence of the *cagPAI* in Hp is associated with an increased risk of severe gastritis, atrophic gastritis, and distal GC compared with strains that lack the *Cag* island [50–52].

### 2.2. Vacuolating Toxin

Hp has the viability of secreting the VacA protein that is modulated by an autotransporter or a secretion pathway of type V [53–55]. This VacA protein has been characterized by its potentiality of causing vacuolation of the cells within the epithelial cortex [56] and is also associated with other immune-suppressive activities [53–55]. The sequence of the amino acid, the effects on the cells, and the structure of the protein are not highly correlated with other strains of toxic bacteria [57]. The alterations at the cells induced by the VacA protein are associated with its potentiality of forming pores in membranes of the host cells [53–55]. The Hp strains have been isolated, with evidence showing that there are variations of the VacA gene and tend to cause different mediated impacts on the cells of the host individual. The description of various VacA alleles has been carried out with reference being given on the variations in the specific regions designated as s, i, and m [58–60]. The most active types in essays of cell-vacuolating activity are the types s1/i1/m1 and have high activity rates compared with those designated s2/i2 or m2 [58, 59, 61, 62]. The variations in the activity of the s1 and s2 types of VacA are associated with the impairment of the channels that characterize the properties of the type s2 protein [61]. Strains that contain VacA alleles with the classification designated “1” are known to encode the active forms of VacA and are also found out to have a higher risk of exposure to GC or premalignant lesions like the metaplasia in the intestines compared with those designated as “2” [59, 63–65].

### 2.3. External Membrane Proteins and DUPA

The Hp genome is known to be made up of nearly 60 genes that are known to encode the membrane proteins at the outer layers. The most extensively researched outer membranes BabA and SabA act as adhesins that have the potential of mediating the Hp that is bounded to the cells at the gastric epithelium. BabA binds to the fucosylated Lewis b histoblood section of the antigen in the cells of the host animal, while SabA has the potential to bind to the sialyl Lewis glycosphigolipid that has metrics [66, 67]. Research has shown that whenever Hp strains cause infection while being bounded to the in-frame babA or sabA genes, there are high risks of development of GC with the alterations in the malignant tumor and the enhancement in the levels of inflammation of the gastric gland compared with the infections that are not bounded to these genes [68–71]. The other strains of OMPs, including the HomB, HopQ, and HopH, are also associated with the development of GC. The first two have a close association with the individual strains containing one or both of the genes that are in correspondence [72].

The other essential gene that has been studied is the dupA or the duodenal-ulcer-promoting gene, which is found in the nonconserved region of the Hp chromosomes known as the region with plasticity and is known as the marker for the GC risk. The strains that harbor such genes are highly associated with the reduction of the atrophy of the gastric gland compared with those genes that do not have the gene [73, 74]. The correlations between the specific OMPs and dupA with GC lack current forms of reported detection compared with the associations between the cagPAI and vacA and GC [34].

## 3. Cytokine Gene Polymorphisms and Gastric Cancer Association

Oncogenic microbes introduce alteration in gastric mucosal homeostasis. Hp is one of the potential microbes known to trigger dysregulation of inflammasome towards the progression of tumorigenesis. Hp-derived carcinogenic or oncogenic soluble effector molecules are associated with modulating inflammatory cytokines aberrantly. Hp-induced perturbation in cytokine gene polymorphism may be implicated in the detection and screening of precancerous suspected patients. In a most recent study, there are several reports available to validate cytokine gene variants in Hp infected patients [75].

## 4. Polymorphisms and *Helicobacter pylori*-Associated Gastric Cancer

Genes that encode cytokines are molecules that are associated therein tend to be comprised of the polymorphic regions that have the potential of altering the transcriptions of the genes, which in turn influences the process of inflammation in response to the infectious diseases [76, 77]. Receptor antagonist genes in humans, including interleukin 10 (*IL-10*), tumor necrosis factor-*α* (*TNF-α*), interleukin-1*β* (*IL-1β*), and interleukin 1 (*IL-1)*, undergo polymorphisms, which in turn influences the expression of cytokines. For instance, in the *IL-10* promoter, single polymorphism of single nucleotides at the 1082 (G/A) positions, the −819 (C/T) position, and the −592 (C/A) position from the transcription start sites have been shown to produce the parent haplotypes, including GCC, ACC, and ATA [78]. The most recent research has shown that SNPs in the TGF*β* −509 C/T, rs1800469, and IL-10 (−819C/T, rs1800871) promoters are known to have a lower risk of GC among samples of participants collected from Mexico [79]. Polymorphisms at the cytokine gene have the power of influencing cytokine expression at the mucosa, gastric inflammation, and the long-term development of lesions, specifically the precancerous. Host polymorphisms are also associated with different types of bacteria, which is an indicator of the host-specific colonization and adaptation. The findings have been useful as they enhance the system of understanding of the complex interplay existing between the host and the bacterial factors engaged in the development of gastric pathology [80].

## 5. Interleukin-10-1082 (*IL-10-1082*), 10-592 (*IL-10-592*), and 10-819 (*IL-10-819*) Promoter Polymorphism and Gastric Cancer Association

The interleukin-10-1082 (*IL-10-1082*), interleukin-10-592 (*IL-10-592*), and interleukin-10-819 (*IL-10-819*) promoter polymorphisms have been found out to be closely associated with GC reported among Asian populations, while the variations in the distribution of genotypes are also associated with the position and Laurenʼs classification of GC [81–83]. The promoter polymorphism defined by *IL-10-1082* is also associated with GC among the Han patients in China, and the variations in the distribution of the genotype are also associated with the position and stage of the GC [84]. In the prognosis of advanced GC, Liu et al. have shown that *IL-10* gene promoter polymorphisms do not act as the determinant factor, and this was established through studies targeting genotype and frequencies of the allele of *IL-10* promoter single nucleotide polymorphism [85]. The most recent research has portrayed the contributory and the susceptibility of GC for the IL-10-592A > C polymorphism and, specifically, among the Asian populations. In contrast, the IL-10-819T > C polymorphism was not found to harbor a high risk of GC. Moreover, large, well-designed research has been called upon to help document the impacts of the IL-10-592A > C and −819T > C polymorphisms on GC [86]. Studies targeting the Iranian populations have also shown how G-197A polymorphism is involved in the interleukin-17 promoter region to GC [87]. The researchers showed that the carriage of a single or more than one G-197A polymorphisms had a significant influence in terms of increasing the GC risks among the patient population. We present the importance of the relationships between the frequencies of variant genotypes of some relevant SNPs and premalignant gastric lesions [75] as shown in Table 1.

Recent studies on the involvement of Hp on GC development through the G-197A polymorphism in *IL-17* have already been established by Rafiei et al. [87], as shown in Table 2.

## 6. Gene Polymorphisms in Cytokines and Toll-Like Receptors and Gastric Cancer Association

Toll-like receptors (TLRs) belong to the massive family of pattern recognition receptors (PRRs), and their activation results in the induction of inflammatory cytokines, chemokines, antigen-presenting molecules, and costimulatory molecules. Recent studies have centered on knowing the association between TLRs and Hp-related diseases [94].

Both TLR2 and TLR were associated with Hp LPS recognition, with contradictory results most likely because of both the inability to obtain pure LPS in experimental studies and the heterogeneity of the bacterial LPS. Furthermore, TLR2 was found to be the foremost extensively expressed gene among all the TLRs in gastric tumors. High levels of TLR4 were conjointly related to a higher risk of GC. TLR5 was initially associated with the recognition of Hp flagellin; however, it looks that this bacterium has developed mechanisms to flee this recognition representing a vital issue concerned with the persistence of this infection and subsequent carcinogenesis. TLR9, the sole TLR with each anti- and proinflammatory role, was concerned with the recognition of Hp DNA. The divided role of TLR9, which promotes or suppresses Hp infection, depends on the stomach environment. Recently, TLR7 and TLR8 were found to recognize purified Hp RNA, thereby inducing proinflammatory cytokines. TLR1 and TLR10 gene polymorphisms were related to a greater risk for GC in Hp-infected individuals. Different gene polymorphisms of these TLRs were found to be related with GC depending mostly on ethnicity. Further studies are needed so as to develop preventive and therapeutic strategies against Hp infections based on the functions of TLRs [94]. The study participants with polymorphism of TLR-4 Thr399 Ile possess an increase in the susceptibility to GC (*P*=0.006), but no significant association for TLR-4 Asp299Gly polymorphism was found [95]. The other polymorphisms, including *TNF-A-308G/A*, *IL-8-251A/T*, *TNF-B* + *252A/G*, and *TLR4* + *1196C/T*, were found to be closely associated with the risks of development of the gastric lesions [96]. However, the association with the increased risk of GC has been documented specifically for the polymorphisms *IL-1RNL/2* with probabilities (*P* < 0.001).

## 7. Prevention of Gastric Cancer

Although the absolute number of GC has remained constant in the last century due to world population growth, the overall decline in incidence continues to support the role of lifestyle, diet, drug use, and other environmental factors in gastric malignancies [97]. GC prevention has concentrated on methods for primary and secondary prevention. Improving diet and lifestyle habits such as reducing the consumption of salty foods, increasing the intake of fruits, and vegetables, smoking cessation and avoiding high alcohol beverages, improving sanitation and hygiene, and reducing the prevalence of Hp, the key cause of GC, maybe the primary prevention strategies for GC at a population level [98]. High levels of vitamins with antioxidant properties and anticancer activities, such as ascorbic acid, carotenoids, and catechins [99], may have had a beneficial effect on the consumption of fruit and vegetables. Interventions for population-level primary prevention can potentially involve very large numbers of individuals that need compelling proof of efficacy. However, in large-scale randomized clinical trials evaluating their effect on the incidence or mortality of GC, interventions based on these dietary methods have not yet been tested [100]. Secondary prevention typically refers to the early detection and treatment of GC using available tools, primarily the endoscopic method [101]. The common application of the nonsteroidal anti-inflammatory drugs (NSAIDs) that prevent GC among patients with peptic ulcers, especially in Hp infected subjects, has been reported in a Taiwanese cohort study using multivariate analysis [102]. Recent studies on mifepristone revealed its therapeutic effects on most of all types of cancer cells. Its pharmacokinetic action involves growth inhibition of various cancer cell lines, suppression of invasive and metastatic cancer potential downregulation of multiple key cellular proteins such as Bcl-2, Cdk2, and NF-Kappa B, cell migration, and interference of heterotypic cell adhesion to basement membrane [103]. In the future, mifepristone may be a wonderful chemopreventive drug, and many studies on this drug are going on across the world. Nowadays, green tea has become a miracle element for many ailments. Hu et al. have reported the curative effects of green tea polyphenols (GTPs) on carcinogenesis. The polyphenols, especially epigallocatechin-3-gallate (EGCG), have the potential of inducing apoptosis, arresting the cell cycle, and suppressing metastasis in the tumor cells, as shown in *in vitro* studies [104]. The green tea polyphenols (GTPs/EGCG) have given promising results on cancer treatment [104]. Serum pepsinogen 1 (PG1) and Hp serology are important tools for the risk of GC stratification in Asia. While testing the sera of Finnish male smokers from the Alpha-Tocopherol, Beta-Carotene Cancer Prevention Study, Song, et al. [105] also showed the usefulness of these markers in the Finnish population. One of the most recent studies underlines that cure of Hp infection should be attempted at any time and rejects the conventional concept of the “point of no return.” It shows a strategy of screening of combined colorectal cancer and gastric preneoplasia at the age >50 years still in time for an effective GC prevention strategy [106].

The GastroPanel® (GP) test (a biomarker panel focused on simultaneous analysis of PGI, PGII, gastrin-17, and Hp IgG antibodies) is the first noninvasive diagnostic test for stomach health [107–109]. GP is not a test for invasive GC, as previously stated [108–110], but rather a screening tool for people who are at risk for GC, such as those who have Hp infection and atrophic gastritis. To date, however, GP has received less validation in population-based screening of such risk groups [111–113].

A recent research supports the use of a panel test that combines pepsinogen, gastrin-17, and anti-Hp antibodies serum assays for screening subjects or populations to identify individuals that are very likely to have atrophic gastritis and should be referred to endoscopy. However, a cost-effectiveness study is needed to assess the role of this test in reduction of GC mortality screening programs. This test may be useful in screening programs aimed at lowering GC mortality. Due to the use of both atrophic gastritis biomarkers, the panel test appears to have a higher sensitivity and specificity than serum pepsinogens and gastrin-17 tests alone [114].

In some previous studies [115–117], it has been found that, on the basis of the concept of “point of no return,” the elimination of Hp cannot reduce the metachronous risk of GC if the gastric mucosa is already damaged via atrophic gastritis/intestinal metaplasia [118]. According to the published clinical studies, Hp eradication and aspirin use are likely to stop the development of metachronous gastric cancer (MGC). Meta-analyses and RCTs show that Hp eradication reduces the risk of MGC development in patients who were followed up for a long term after the eradication (≥5 years) [119].

## 8. Conclusion

Hp has been shown to be a gastric bacterial pathogen known to result in the colonization of more than half of the population across the world. Hp infection is known to provoke chronic inflammation and the increase in the likelihood of progression of the duodenal and gastric ulcer disorder and GC [120]. GC has also been documented as one of the most complex disorders that are a consequence of the interaction of multiple processes, including factors like lifestyle, dietary habits, and infections, alongside the variants of genes and molecular alterations that may accrue along the developmental stages of an individual. The published research provides evidence on the interactions between the host and Hp as the unveiling of the colonization of the human stomach and different associated characteristics tend to contribute to the infection outcomes. The host cytokines gene polymorphisms and the virulence of Hp factors in GC progression have been documented as essential factors in determining the prognosis of the patient [121]. The understanding that GC results from Hp-induced chronic inflammation suggested that eradication of Hp would also eliminate GC and other Hp-related diseases. This realization is additionally forcing current GC prevention programs emphasizing early detection to also consider GC prevention via eradication of the causative agent. For low or moderate risk groups, Hp eradication alone is perhaps the foremost cost-effective strategy, whereas high-risk groups, identified based on the extent and severity of atrophic damage, are likely to take advantage of continued surveillance [122]. We believe that, within the next five years, many regions and countries will consider or implement Hp eradication programs that may vary from mass population-based Hp eradication in countries with high risk to limited programs in countries with low risk, which are tailored to specific high-risk groups. In countries with high risk, the new programs will either integrate or more likely replace current programs. Planning should address (a) Who the candidates are? (b) Can the program be implemented cost-effectively? and (c) How to prioritize the program with respect to other healthcare programs and in reference to the use of the nationʼs resources? A variety of pilot programs are already underway. In high Hp prevalence countries with low gastric cancer burdens, preventive or curative vaccination would likely be the sole cost-effective alternative such that developed countries should support vaccine development [122]. Cleansing the world of Hp infection by vaccination (when it becomes available) or globally applying eradication strategies is currently a priority [123, 124].

## Figures and Tables

**Table 1:** Association between frequencies of some important cytokine SNP genotypes and premalignant gastric lesions.

SNP	Variant genotypes	Variable genotype frequency (%) in premalignant gastric lesions vs. control	Premalignant gastric lesions	References
TNF-*α*-308	GA AA	15 (29.4)/22 (20.4) 0 (0)/3 (2.8)	Chronic atrophic gastritis susceptibility	[88]
IL-1B-511	CT TT	18 (35.3)/53 (49.1) 3 (5.9)/13 (12.0)	Chronic atrophic gastritis susceptibility	
IL-1RN	L/2 2/2	15 (29.4)/32 (29.6) 2 (3.9)/13 (12.0)	Chronic atrophic gastritis susceptibility	

TNF-*α*-308	GA AA	125 (23.4)/129 (24.2) 13 (2.4)/8 (1.5)	Chronic atrophic gastritis susceptibility	[89]
IL-10 T-819C	TC CC	200 (37.5)/199 (37.3) 44 (8.2)/28 (5.2)	Chronic atrophic gastritis susceptibility	
IL-10 A-1082G	AG GG	246 (46.1)/262 (49.1) 109 (20.4)/116 (21.7)	Chronic atrophic gastritis susceptibility	

IL-10-1082	AA GA	35 (60.3)/81 (49.1) 17 (29.3)/56 (33.9)	Atrophic gastritis risk	[90]
	AA GA	17 (54.8)/99 (51.6) 10 (32.3)/63 (32.8)	Corpus atrophic gastritis risk	
	AA GA	12 (52.2)/104 (46/6) 10 (43.5)/63 (28.3)	Intestinal metaplasia risk	
IL-10-592	AA CA	8 (13.8)/12 (7.3) 19 (32.8)/58 (35.1)	Atrophic gastritis risk	
	AA CA	4 (12.9)/16 (8.3) 10 (32.3)/67 (34.9)	Corpus atrophic gastritis risk	
	AA CA	4 (17.4)/16 (7.2) 8 (34.8)/69 (30.9)	Intestinal metaplasia risk	

IL10–1082	AG AA	102 (35.3)/406 (37.5) 142 (49.1)/508 (46.9)	Atrophic gastritis risk	[91]
	AG AA	201 (37.0)/406 (37.5) 274 (50.5)/508 (46.9)	Intestinal metaplasia risk	
	AG^a^ AA^a^	51 (43.2)/406 (37.5) 60 (50.8)/508 (46.9)	Dysplasia risk	

IL-10-1082	AG GG	15 (12.9)/18 (7.8) 0 (0)/1 (0.4)	Atrophic gastritis susceptibility	[92]
IL-10-819	CT CC	47 (40.5)/104 (44.8) 28 (24.1)/36 (15.5)	Atrophic gastritis susceptibility	
IL-10-592	AC CC	46 (39.7)/96 (41.4) 31 (26.7)/52 (22.4)	Atrophic gastritis susceptibility	

IL-10-819	CT TT	55 (41.9)/21 (55.3) 10 (7.6)/6 (15.8)	Gastritis	[93]
IL-10-819	CT TT	21 (42.9)/21 (55.3) 5 (10.2)/6 (15.8)		

Relative frequencies (%) are mentioned in relation to the size of each of the two groups (cases and control group); ^a^*P* < 0.05.

**Table 2:** Association between *G-197A* polymorphism and gastric cancer development *n* (%).

Group/category	GA and AA	GG	Odds ratio	95% CI	*P* value^2^
*Age*					
Less than 50 years	18 (17.1)	4 (7.1)	2.7	0.8–8.4	0.09
50 and more than 50 years	87 (82.9)	52 (92.9)			

*Gender*					
Male	51 (48.6)	21 (37.5)	1.6	0.8–3.05	0.19
Female	54 (51.4)	35 (62.5)			

*Helicobacter pylori*					
Positive	65 (61.9)	33 (58.9)	0.88	0.45–1.71	0.74
Negative	40 (38.1)	23 (41.1)			

*Tumor node metastasis stage^1^*					
I-II	22 (55)	6 (16.2)	6.3	2.2–18.5	0.001
III-IV	18 (45)	31 (83.8)			

*Tumor differentiation*					
High	25 (23.8)	9 (16.1)	1.00^3^		
Average	57 (54.3)	35 (62.5)	0.56	0.24–1.34	0.19
Poor	23 (21.9)	12 (21.4)	0.66	0.23–1.86	0.43

^1^Data conferred for 77 patients. ^2^Comparisons between categorical variables were done using a two-sided *χ*2 test. ^3^Used as a control for tumor differentiation analyses. Characters/values in parentheses show the percentage.
